# Generalized mathematics self-efficacy and student engagement: the role of self-concept and mathematics anxiety

**DOI:** 10.3389/fpsyg.2026.1852588

**Published:** 2026-06-15

**Authors:** Erik Bergqvist

**Affiliations:** Department of Health, Education and Technology, Division of Education and Languages, Luleå University of Technology, Luleå, Sweden

**Keywords:** bayesian statistics, generalized mathematics self-efficacy, mathematics anxiety, mathematics self-concept, path analysis, problem-solving, student engagement

## Abstract

**Introduction:**

Mathematics self-efficacy and mathematics self-concept are well-established predictors of student engagement and performance. However, their relative contributions, alongside mathematics anxiety, are rarely examined within a single model. This study investigates how generalized mathematics self-efficacy, mathematics self-concept, and mathematics anxiety are associated with multiple dimensions of student engagement in the context of collaborative work on mathematical problems.

**Methods:**

Seventy-seven Swedish upper-secondary students participated in a 2-week instructional segment involving complex problem-solving, with data collected at two time points. Associations among mathematics self-beliefs, mathematics anxiety, and engagement were examined using linear mixed-effects models within both frequentist and Bayesian frameworks.

**Results:**

The results indicate that generalized mathematics self-efficacy shows consistent positive associations with all dimensions of engagement, whereas mathematics self-concept displays more limited and selective relations. Mathematics anxiety is primarily associated with cognitive disengagement. In addition, patterns of associations suggest that prior achievement may relate to engagement through its relation to generalized mathematics self-efficacy.

**Discussion:**

These findings are consistent with theoretical perspectives emphasizing the role of control-related beliefs and achievement emotions in shaping engagement. The study highlights the importance of distinguishing between different types of mathematics self-beliefs and emotional processes and demonstrates the value of examining them jointly within a single analytical framework.

## 1 Introduction

Understanding students' domain-specific self-beliefs remains a central challenge in educational psychology, partly due to persistent conceptual and measurement ambiguity. A particularly important issue concerns the distinction between mathematics self-efficacy and mathematics self-concept. These constructs are frequently conflated, partially overlapping, or inconsistently operationalized across studies, which has contributed to well documented jingle jangle fallacies, where similar labels refer to different constructs or different

labels refer to essentially the same construct (Bong and Skaalvik, 2003; Marsh et al., 2019; Lee et al., 2020). Such ambiguity weakens construct validity and complicates theory development and empirical interpretation in motivation research (Pekrun, 2024).

Despite these challenges, mathematics self-efficacy and mathematics self-concept are consistently identified as important predictors of student engagement and academic behavior (Zimmerman, 2000; Linnenbrink and Pintrich, 2003). Although some empirical work suggests limited differentiation between domain-specific generalized mathematics self-efficacy, as termed in this study, and mathematics self-concept (Marsh et al., 2019), their theoretical foundations may reflect distinct self-beliefs. For instance, generalized mathematics self-efficacy reflects a self-belief that emphasizes a more future-oriented perception of capability, whereas mathematics self-concept reflects a broader evaluative and more past-oriented self-perception (Marsh et al., 2019). These differences suggest that the two constructs may influence engagement in distinct ways. However, few studies have examined these self-beliefs simultaneously within a single analytic framework that allows for clear differentiation and direct comparison.

Mathematics anxiety is conceptually distinct from self-beliefs. However, at the same time, it is closely related to perceived competence and control, suggesting potential interactions with mathematics self-beliefs in shaping engagement. Mathematics anxiety is widely recognized as a proximal determinant of engagement and learning, as it can undermine students' perceived competence and sense of control (Pekrun, 2006). Moreover, emotional components are sometimes embedded within measures of mathematics self-concept (Klee et al., 2022), and high correlations between anxiety and self-belief measures often lead to extensive multicollinearity, complicating the identification of their unique associations with engagement. For example, (Pajares and Miller 1994) reported correlations so high that mathematics anxiety could not be retained in their analyses. However, more recent work suggests that careful measurement and attention to multicollinearity prior to factor-analytic modeling can allow for clearer empirical differentiation among mathematics anxiety, generalized mathematics self-efficacy, and mathematics self-concept (Bergqvist, 2024).

Student engagement itself is shaped by both individual beliefs and situational characteristics of the learning environment. Cognitive engagement often varies across instructional contexts, particularly during problem-solving and algorithmic activities (Liu et al., 2023; Xing et al., 2023). Moreover, students' perceptions of their mathematical competence can fluctuate over short time spans depending on task difficulty and instructional demands (Street et al., 2022). In addition, direct comparisons of generalized mathematics self-efficacy and mathematics self-concept within the same empirical model remain rare, leaving important questions about their conceptual boundaries and differential predictive value unresolved.

Addressing this gap, the present study examines how generalized mathematics self-efficacy and mathematics self-concept are associated with student engagement in the context of collaborative work on mathematical problems, while considering the role of mathematics anxiety, prior achievement, and gender.

Collaborative problem-solving provides an instructional context that involves peer interaction, shared regulation of learning, and opportunities for social comparison, which may shape how students perceive their competence and respond emotionally during mathematics learning. Accordingly, collaboration is treated as the instructional setting in which engagement unfolds, rather than as a construct of the present study. This implies that the present study does not aim to model collaborative processes *per se* but rather examines how mathematics self-beliefs and mathematics anxiety are associated with engagement within this instructional context.

## 2 Theoretical framework

### 2.1 Mathematics self-beliefs

Mathematics self-concept refers to individuals' perceptions of their own abilities in learning mathematics, performing well in mathematics classrooms, and achieving strong results on mathematics assessments. These perceptions are shaped both by personal evaluations of competence and by the feedback they receive from others. Mathematics self-concept also contributes to broader self-perceptions by informing how individuals compare their mathematical abilities with their abilities in other academic domains (Shavelson et al., 1976; Reyes, 1984; Marsh and Shavelson, 1985).

Self-efficacy theory differentiates between beliefs about one's capability to perform specific tasks and more generalized judgments that lack explicit performance criteria. Task-specific mathematics self-efficacy refers to confidence in completing clearly defined mathematical activities (e.g., “I am confident that I can solve this equation”), whereas generalized mathematics self-efficacy reflects a broader, non-task-specific sense of capability without reference to concrete benchmarks (Marsh et al., 2019). Such generalized judgments often involve social comparison, as exemplified by items like “I believe I can do well on math tests.” Accordingly, generalized mathematics self-efficacy is conceptually similar to mathematics self-concept (Marsh et al., 2019). Nevertheless, the constructs are treated as distinct in the present study since mathematics self-concept primarily reflects retrospective, experience-based evaluations of overall mathematical ability (e.g., “I believe I am the kind of person who is good at mathematics”), whereas generalized mathematics self-efficacy represents a future-oriented belief in one's capability to succeed in mathematics without reference to specific tasks (e.g., “I believe I can understand the content in a mathematics course”).

A large body of research demonstrates that the predictive power of self-efficacy beliefs depends on the correspondence between the belief and the outcome being measured (Pajares, 1996; Bong and Skaalvik, 2003). In mathematics, task-specific self-efficacy more strongly predicts performance on specific tests, while generalized self-efficacy shows stronger associations with broad academic indicators such as overall mathematics grades or long-term achievement (Pajares, 1996; Bandura, 2005; Marsh et al., 2005). Distinguishing these forms of self-belief is therefore essential for interpreting inconsistent findings in the literature concerning the relative predictive strength of self-efficacy and self-concept for academic achievement (Byrne and Shavelson, 1986; Pajares and Graham, 1999; Marsh et al., 2005).

### 2.2 Mathematics anxiety

Mathematics anxiety is conceptually distinct from mathematics self-beliefs and is therefore treated as an achievement emotion rather than a competence-related belief. Whereas self-efficacy and self-concept reflect perceived competence, mathematics anxiety captures affective responses that arise in mathematical situations. This distinction aligns with Control-Value Theory (Pekrun, 2006), which conceptualizes achievement emotions as shaped by appraisals of perceived control and subjective value, and as proximal antecedents of engagement. In this perspective, mathematics anxiety is understood as an emotional response linked to students' perceptions of competence and control, rather than as a belief. Accordingly, mathematics anxiety is included alongside mathematics self-beliefs in the present study while remaining conceptually distinct from them.

Mathematics anxiety can be described as a feeling of tension or apprehension associated with performing mathematical tasks (Zeidner and Matthews, 2005). It is broader than fear, which refers to reactions to a clearly identifiable threat, and may interfere with problem-solving by consuming working memory resources needed for effective reasoning (Ashcraft, 2002). Anxiety may be experienced as a relatively stable trait or as a situation-specific state that fluctuates over time (Zeidner and Matthews, 2005; Pekrun, 2006). Research on mathematics anxiety has shown consistent links to avoidance behavior, reduced engagement, and impaired working memory during problem-solving (Ashcraft, 2002; Hembree, 1990). These mechanisms are particularly relevant in demanding mathematical tasks, where anxiety may interfere with cognitive processing and reduce persistence. Both forms are relevant for motivational processes, as they can influence students' willingness to engage with mathematics and their persistence when facing challenges.

### 2.3 Student engagement as a multidimensional construct

In educational settings, engagement refers to how actively involved students are in their learning activities (Connell and Wellborn, 1991; Fredricks et al., 2004; Skinner et al., 2009). It is often viewed as a reflection of their motivation to learn (Connell and Wellborn, 1991; Ryan and Deci, 2020). Researchers agree that student engagement is complex, but its understanding can vary depending on the context (Fredricks et al., 2004; Appleton et al., 2008). However, it is commonly viewed as involving behavioral (actions), emotional (feelings), and cognitive (thinking) aspects, as well as its opposite, disengagement (Connell and Wellborn, 1991; Skinner et al., 2009). While these dimensions can be considered separately, they are closely connected to each other (Fredricks et al., 2004; Skinner et al., 2009; Jang et al., 2016). Student engagement represents the visible outcomes of student motivation, as highlighted by (Connell and Wellborn 1991) and (Ryan and Deci 2020). Conversely, students' views on their own mathematical abilities and their anxiety about mathematics are personal and often implicit, as discussed by (Schunk and Pajares 2005). However, it is important to acknowledge the deep connection between these two aspects and their significant impact on student motivation, as emphasized by (Skinner et al. 2008) and (Skinner et al. 2009).

Behavioral engagement refers to the extent of effort and persistence a learner invests, coupled with mental processes like concentration and attentiveness. Emotional engagement involves feelings of interest, joy, and enthusiasm. Conversely, disaffection, its opposite, manifests as behavioral passivity and withdrawal, alongside emotional states of frustration, anxiety, and boredom (Skinner et al., 2008, 2009). Cognitive engagement involves the use of learning strategies that promote deep comprehension, in contrast to surface-level understanding (Connell and Wellborn, 1991; Reeve, 2018). Cognitive disengagement, on the other hand, is characterized by the use of disorganized learning strategies (Elliot et al., 1999; Jang et al., 2016). According to Reeve (2013), “agentic engagement can be viewed not just as a student's contributions into the flow of instruction but also as an ongoing series of dialectical transactions between student and teacher” (p. 580). For instance, agentic engagement is evident in the classroom when students take initiative by asking questions, offering helpful feedback to improve the learning environment for everyone, and expressing their educational needs and goals (Reeve and Tseng, 2011; Reeve, 2012).

### 2.4 Theoretical perspectives on self-beliefs, emotions, and engagement

Student engagement and mathematics self-beliefs are shaped by both individual and contextual factors and should therefore be understood within the dynamics of the classroom. The Self-System Model of Motivational Development (SSMMD), originally developed by (Connell and Wellborn 1991) and further elaborated by Skinner and colleagues (Skinner and Belmont, 1993; Skinner et al., 2008, 2009), provides a theoretical framework that situates self-beliefs within a broader motivational system. This system, for example, links mathematics-related self-beliefs with student engagement. Building on this perspective, recent work has emphasized the value of integrating complementary motivational frameworks to better capture the dynamic interplay between classroom context, self-beliefs, and student engagement (Fryer and Leenknecht, 2023). Moreover, the SSMMD framework helps explain the cyclical interplay between classroom context (e.g., teacher instruction) and student engagement (Reeve et al., 2022; Jang et al., 2024).

This framework is particularly effective for clarifying motivational processes in educational settings because it explicitly links constructs such as self-concept and self-efficacy to observable engagement behaviors. While Self-Determination Theory (SDT) emphasizes that the quality of motivation depends on fulfilling basic psychological needs for competence, autonomy, and relatedness (Ryan and Deci, 2020), SSMMD extends this principle by demonstrating how competence-related beliefs, including self-concept and self-efficacy, directly influence engagement in classroom contexts (Skinner and Belmont, 1993). This emphasis makes SSMMD appropriate for research examining the predictive role of self-beliefs in student engagement, as it offers a classroom-oriented explanation of the motivational mechanisms underlying student engagement (Fryer and Leenknecht, 2023).

To further clarify the role of emotions within this system, the present study also draws on Control-Value Theory (CVT; Pekrun, 2006). According to CVT, achievement emotions arise from appraisals of perceived control and subjective value and function as proximal antecedents of engagement. Within this perspective, mathematics self-beliefs can be understood as reflecting control-related appraisals, whereas mathematics anxiety represents an achievement emotion emerging from these appraisals.

According to SSMMD, motivation creates a feedback loop that drives the process forward. For example, an instructor's supportive and adaptable teaching methods can enhance students' motivation to learn (Reeve and Shin, 2020; Jang et al., 2024). This, in turn, facilitates the acquisition of knowledge and skills, potentially sparking interest and encouraging the use of effective learning strategies (cognitive engagement). These strategies may in turn lead to an increase in student-generated questions and active involvement (agentic engagement), thereby fostering a more supportive teaching environment, provided that the instructor responds positively (Tsai et al., 2008). This cyclical process consequently influences changes in student engagement (Reeve et al., 2022; Jang et al., 2024).

Based on the theoretical perspectives outlined above, the present study assumes a conceptual model in which mathematics self-beliefs (mathematics self-concept and generalized mathematics self-efficacy) are associated with students' emotional experiences (mathematics anxiety), which in turn are related to different dimensions of student engagement. In addition, prior achievement and gender are included as antecedent variables associated with self-beliefs, emotions, and engagement. These relations are expected to be interrelated and potentially reciprocal but are examined here as associational patterns.

## 3 Empirical associations with student engagement

The following section reviews prior empirical findings related to the variables included in the present study.

### 3.1 Mathematics self-beliefs and student engagement

Previous research has consistently demonstrated strong associations between mathematics self-efficacy and self-concept beliefs and student engagement (Linnenbrink and Pintrich, 2003; Patrick et al., 2007; Sakiz et al., 2012; Martin and Rimm-Kaufman, 2015; Wang et al., 2017). Beliefs about perceived competence, including mathematics self-concept and self-efficacy, together with contextual factors, play a critical role in shaping engagement (Zimmerman, 2000; Linnenbrink and Pintrich, 2003). Within the SSMMD, these self-beliefs are central to self-system processes that mediate the influence of contextual supports on engagement.

### 3.2 Mathematics anxiety and student engagement

The relationships among students' generalized mathematics self-efficacy, mathematics self-concept, and mathematics anxiety are well-established in prior research (Hembree, 1990; Pajares and Miller, 1994; Pajares and Kranzler, 1995; Goetz et al., 2010). Previous studies have repeatedly demonstrated a negative association between mathematics anxiety and student engagement, particularly behavioral and cognitive engagement (e.g., Archambault et al., 2022). However, distinctions between state and trait anxiety have been identified when task difficulty and learning context are considered (Guo and Liao, 2025). Nonetheless, it remains an open question whether mathematics anxiety mediates the effects of generalized mathematics self-efficacy and mathematics self-concept on student engagement.

### 3.3 Prior achievement and student engagement

Self-concept-like constructs, such as generalized mathematics and mathematics self-concept, are strongly associated with prior mathematics achievement (Holenstein et al., 2022; Bergqvist, 2024). Research has further demonstrated a reciprocal relationship between domain-specific self-concept (e.g., mathematics self-concept) and achievement, whereby earlier self-concept influences later performance and prior achievement shapes subsequent self-concept development (Marsh, 1990; Marsh and Yeung, 1997). Indeed, considerable evidence in the literature supports self-concept as both a cause and an effect of performance, as highlighted by (Wu et al., 2021). Consequently, prior mathematics achievement may influence student engagement through its impact on mathematics-related self-beliefs.

### 3.4 Gender and student engagement

Gender disparities in personal beliefs within educational settings have been extensively documented. Research indicates that females often exhibit lower levels of efficacy (Zander et al., 2020), decreased confidence in their overall competence (Skaalvik and Skaalvik, 2004), and higher levels of anxiety compared to their male counterparts (Pajares and Kranzler, 1995; Devine et al., 2012; Xie et al., 2019; Morán-Soto and González-Peña, 2022; Bergqvist, 2024). However, it is crucial to acknowledge that these differences may be subject to the influence of social contexts (Hyde, 2005; Else-Quest et al., 2010). Indeed, the impact of gender variations on personal beliefs may diminish or vanish altogether when additional variables, such as prior academic achievement, are considered (Pajares, 2005).

Further, studies have consistently found that females demonstrate elevated levels of behavioral and emotional engagement in comparison to their male counterparts within educational settings (Furrer and Skinner, 2003; Skinner et al., 2008; Lietaert et al., 2015; Hofer et al., 2022). Moreover, a subset of research posits that the observed gender discrepancy in behavioral engagement may, in part, be explicable through the provision of autonomy support by teachers (Lietaert et al., 2015). However, previous research presents inconclusive findings regarding gender differences in cognitive engagement. Some studies suggest that, when controlling for prior personal goal orientation, there are no significant gender differences in cognitive engagement (Young, 1997). However, other research indicates that female students may exhibit higher levels of cognitive engagement, particularly in their use of strategies (Zimmerman and Martinez-Pons, 1990; Patrick et al., 1999; Fredricks et al., 2005).

## 4 Aim and research questions

The aim of this study is to examine how generalized mathematics self-efficacy and mathematics self-concept relate to students' engagement in the context of collaborative work on complex, realistic mathematical problems. In doing so, the study compares the relative contributions of generalized mathematics self-efficacy and mathematics self-concept, while also considering the potential role of mathematics anxiety, prior achievement, and gender.

The research questions are as follows:

- What are the relative contributions of mathematics self-concept, generalized mathematics self-efficacy, and mathematics anxiety to the prediction of different dimensions of student engagement?
- To what extent do generalized mathematics self-efficacy, mathematics self-concept and mathematics anxiety differ in mediating the effects of prior achievement and gender on students' engagement?

To address the research questions, data were collected using a questionnaire consisting of Likert-type scales. The analyses focus on patterns of associations among mathematics self-beliefs, mathematics anxiety, and engagement that are consistent with a control-emotion-engagement sequence. In this context, mediation-related questions are examined in an exploratory manner, focusing on patterns of associations. These patterns are interpreted as consistent with indirect relationships rather than establishing causal pathways. The following sections describe the method and present results from an analysis of the interplay between generalized mathematics self-efficacy, mathematics self-concept, and dimensions of student engagement.

## 5 Materials and methods

### 5.1 Procedure and data collection

The study was conducted as part of regular mathematics instruction, during a curriculum segment focusing on mathematically rich and contextually realistic problem situations. Participants were 77 students (29 female, 48 male) in their final semester of upper secondary school in Sweden (ages 18–19), enrolled in a university entrance qualification program emphasizing theoretically advanced mathematics. Over a 2-week period comprising seven lessons, students collaboratively worked on a selected problem, with instruction facilitated by the classroom teacher. Participation was voluntary, informed consent was obtained prior to their involvement, and all procedures followed the ethical guidelines of the Swedish Research Council.

The instructional segment began with an introductory task designed to illustrate that mathematical problems may have multiple solutions and to emphasize connections between mathematical problem solving and computational thinking, including decomposition, pattern recognition, abstraction, algorithmic reasoning, testing, and generalization (ISTE, 2011; Selby and Woollard, 2014). Following this introduction, students independently selected a mathematically rich problem from a designated chapter of the course textbook to work on throughout the 2-week period (see examples in Alfredsson et al., 2019). Three class teachers provided ongoing support during the lessons.

Data were collected at two time points (pre- and post-test) during the instructional period. The results are interpreted as reflecting associations across observations rather than changes over time. Time was included as a predictor in subsequent analyses to capture systematic differences between measurement occasions. Accordingly, the repeated observations are treated as pooled data with within-person dependency, rather than as a basis for modeling individual change trajectories.

### 5.2 Measures

Each of the original items was written in English but translated into Swedish. The same five-point Likert-type scale was used throughout the questionnaire, with the following response options: 1 = aldrig (*never*), 2 = sällan (*seldom*), 3 = ibland (*sometimes*), 4 = ofta (*often*), 5 = nästan jämnt (*usually*).

#### 5.2.1 Student engagement

To assess students' behavioral and emotional engagement, the behavioral (e.g., “When I'm in this class, I listen very carefully.”) and emotional engagement (e.g., “This class is fun.”) from engagement vs. disaffection with learning measure (Skinner et al., 2009) were used. In the present study, the behavioral and emotional measures were shown to be internally consistent (Cronbach's α were 0.89/0.89, and 0.87/0.85 in the pre- and post-tests, respectively). However, one item from the original behavioral engagement scale (“When I'm in this class, I participate in class discussions.”) was excluded from the scale due to its weak correlations (approximately 0.30) with other items in the subscale. This exclusion led to enhanced internal consistency for the behavioral engagement measure, as indicated by Cronbach's α in the pre- and post-tests (0.95 and 0.91).

Agentic engagement (e.g., “I let my teacher know what I need and want.”) was assessed using the agentic engagement scale (Jang et al., 2016) and was found to be internally consistent in this study (α = 0.83/0.84). Students' cognitive engagement (e.g., “When reading for this class, I try to explain the key concepts in my own words.”) and disengagement (e.g., “In this course, I often find that I don't know what to study or where to start.”) were assessed using the deep learning (Senko and Miles, 2008; Jang et al., 2016), and study disorganization measures (Elliot et al., 1999; Jang et al., 2016). Both cognitive engagement and disengagement were included, as they capture distinct dimensions of students' learning strategies. In the present study, Cronbach's α were 0.80/0.83, and 0.89/0.83, respectively. The complete wording of all student engagement items is provided in the Supplementary Material (Table 4).

As the engagement items were originally developed in English and subsequently translated into Swedish, a confirmatory factor analysis (CFA) was conducted to examine the factorial structure of the translated instrument. The analysis indicated that a five-factor model, representing behavioral, emotional, agentic, and cognitive engagement, as well as cognitive disengagement, provided an acceptable fit to the data.

Model parameters were estimated using diagonally weighted least squares (DWLS) with polychoric correlations. To address potential small-sample bias in model fit indices, the Swain correction was applied using the R function *swain* (Version 1.2; Boomsma and Herzog, 2013). Based on commonly used cut-off criteria (Hu and Bentler, 1999), the model showed good fit, χ^2^(220) = 241.11, *p* = 0.16 (Swain-corrected χ^2^(220) = 212.44, *p* = 0.63), with a TLI of 0.997 (Swain-corrected TLI 1), and RMSEA 0.04 (Swain-corrected 0), 90% CI [0.00, 0.06], and Swain-corrected CI [0, 0.04]. However, given the limited sample size and model complexity, these fit indices should be interpreted with caution and are reported primarily as descriptive support for the proposed factor structure rather than as strong confirmatory evidence.

#### 5.2.2 Generalized mathematics self-efficacy and mathematics self-concept

To measure students' generalized mathematics self-efficacy (e.g., “I believe I can understand the content in a mathematics course”) and mathematics self-concept (e.g., “I believe I am the kind of person who is good at mathematics”), items presented by Bergqvist (2024) were used. Since these scales evaluate concepts that are often difficult to operationalize clearly, short scales of three items, such as those proposed by Bergqvist (2024), may provide a psychometrically valid alternative to longer scales (Gogol et al., 2014). Although these constructs are conceptually related and may show empirical overlap, they are treated as distinct in the present study to examine their potentially different associations with engagement. In the present study, Cronbach's α were 0.79 and 0.73, and 0.87 and 0.89, respectively, in the pre- and posttests.

#### 5.2.3 Mathematics anxiety

To assess mathematics anxiety, two subscales from Bergqvist (2024) were used: In-class anxiety (e.g., “I am afraid to give an incorrect answer during my mathematics class”) and assignment anxiety (e.g., “Working on mathematics homework is stressful for me”). While most scales showed acceptable internal consistency, the assignment anxiety scale demonstrated lower reliability at post-test (Cronbach's α were 0.82 and 0.90 for in-class anxiety, and 0.75 and 0.51 for assignment anxiety, respectively).

#### 5.2.4 Prior mathematics achievement, gender and background variables

Students were asked to report their legal gender and their most recent grade in mathematics, which served as a measure of their prior achievement in the subject. Prior achievement should therefore be interpreted as an approximate indicator rather than an objective measure of prior performance. In Sweden, mathematics grades range from F (fail) to E, D, C, B, and up to A (highest grade). In addition, students were asked to specify their problem-solving group and the mathematics class they belonged to (identified as A, B, C, or D). This additional information was collected primarily to facilitate the pairing of student responses.

### 5.3 Data analysis

The analyses were conducted in three steps. First, missing data were addressed using multiple imputation, and latent factor scores were subsequently estimated for each construct. Second, associations between variables were examined using linear mixed-effects models to account for repeated observations within individuals. Third, a path-analytic framework was applied to explore patterns of associations among self-beliefs, mathematics anxiety, and student engagement. The analytical strategy should be considered exploratory, and multiple analytical approaches were used to examine the robustness of the findings.

In the Classical Test Theory (CTT) framework, a common method for assessing latent traits involves summing item responses. However, this practice may not adequately represent the underlying constructs due to its reliance on simple linear transformations (Rusch et al., 2017; McNeish and Wolf, 2020). To address measurement challenges, a unidimensional IRT factor model was estimated for each latent variable using the R package *mirt* (Version 1.45.1; Chalmers, 2012). Factor scores were derived from the posterior distributions using the *fscores()* function within the R package *mirt*, applying Maximum A Posteriori (MAP) estimation, and summarized using the median.

Gender, prior mathematics achievement, and latent factors were analyzed using a series of linear mixed-effects models, estimated within both frequentist and Bayesian approaches. Each model included participant as a random effect to account for within-individual dependency and repeated observations (Field et al., 2013). Time (pre-test vs. post-test) was included as a fixed-effect predictor to capture mean-level differences between measurement occasions. This approach allows the inclusion of both observations while accounting for dependency in the data; however, it does not constitute a fully longitudinal design. Accordingly, the estimated relations should be interpreted as associations across observations rather than as within-person change over time.

In the Bayesian analyses, posterior distributions were approximated using Markov Chain Monte Carlo (MCMC) methods, which are suitable for small samples as they do not rely on large-sample assumptions (McNeish, 2016; Miočević, 2020). In addition, Bayesian multivariate models are fitted using the *brm()* function in the R package *brms* (Version 2.23.0; Bürkner, 2017, 2018, 2021), to provide more robust parameter estimates. To minimize bias while allowing the data to inform the results, weakly informative priors were specified. A normal distribution with a mean of zero and a standard deviation of 10 was used for most parameters, while a normal distribution with a mean of zero and a standard deviation of 1 was used for multilevel correlations to reflect the bounded nature of correlation coefficients (McNeish, 2016; Zondervan-Zwijnenburg et al., 2017; Yamamoto and Miyazaki, 2024).

Posterior indices and Region of Practical Equivalence (ROPE) metrics were computed using the R package *bayestestR* (Version 0.17.0; Makowski et al., 2019b). The probability of direction (pd) indicates the proportion of the posterior distribution that shares the same sign as the median, with pd values of 95, 97.5, 99.5, and 99.95 corresponding to frequentist *p*-value thresholds of 0.1, 0.05, 0.01, and 0.001, respectively (Makowski et al., 2019a). ROPE defines a range of values considered practically negligible, and the proportion of the posterior distribution within this range was used to evaluate the practical relevance of effects (Kruschke, 2018).

Missing data were addressed using multiple imputation with a multinomial logit model implemented in the R package *mice* (Version 3.18.0; van Buuren and Groothuis-Oudshoorn, 2011). The imputation model was specified separately for different sets of variables: missing values in mathematics self-belief measures were imputed using other self-belief variables, while engagement variables were imputed using other engagement indicators. A total of five imputed datasets were generated, each based on 50 iterations. The imputation models included between 15 and 25 predictor variables, following recommended practices for multiple imputation (van Buuren and Groothuis-Oudshoorn, 2011).

Finally, a path-analytic approach was used to examine the predictive and mediating roles of generalized mathematics self-efficacy, mathematics self-concept, mathematics anxiety, and dimensions of student engagement. In these models, engagement dimensions were treated as dependent variables, while mathematics self-beliefs, anxiety measures, prior achievement, gender, and time were included as predictors. Time was included as an antecedent predictor in the path model to account for differences between measurement occasions. The directional structure of the model reflects theoretical assumptions rather than empirically established temporal ordering. An overview of the statistical models is presented in Table 1. The table summarizes the outcome variables, predictors, and model specifications used in the analyses. All models include time (pretest vs. posttest) as a predictor and account for repeated observations by incorporating participant-level random intercepts. The estimates are interpreted as associations across observations rather than as evidence of longitudinal or causal relationships.

**Table 1:** Overview of statistical models and model specifications.

Model	Outcome variable	Fixed effects (predictors)	Random effects	Data structure	Estimation
M1	Mathematics self-concept (MSC)	Prior achievement, gender, time	Participant (random intercept)	Repeated observations (pre/post)	Robust linear mixed-effects model; Bayesian estimates for robustness
M2	In-class anxiety (ICA)	Prior achievement, gender, time, MSC	Same as M1	Same as M1	Same as M1
M3	Generalized mathematics self-efficacy (GMS)	Prior achievement, gender, time, MSC, ICA	Same as M1	Same as M1	Same as M1
M4	Assignment anxiety (ASA)	Prior achievement, gender, time, MSC, ICA, GMS	Same as M1	Same as M1	Same as M1
M5-M9	Each dimension of engagement	Prior achievement, gender, time, MSC, ICA, GMS, ASA	Same as M1	Same as M1	Same as M1

All models include repeated observations nested within participants and are estimated with random intercepts to account for within-person dependency. Estimates should be interpreted as associations across observations rather than as evidence of longitudinal change or causal relationships.

The proposed path model was informed by the theoretical framework, particularly the assumption that competence-related self-beliefs reflect control-related appraisals that are associated with achievement emotions, which in turn are related to engagement. Accordingly, the model included paths from mathematics self-concept to generalized mathematics self-efficacy, from mathematics self-beliefs to mathematics anxiety, and from these variables to multiple dimensions of student engagement. Prior achievement, gender, and time were included as antecedent variables associated with self-beliefs, anxiety, and engagement.

## 6 Results

Both the pre- and post-test data contain 12% missing values. Given the small sample size, listwise deletion would substantially reduce statistical power (Brown, 2015). Therefore, missing values are handled using multiple imputation with a multinomial logit model implemented via the *mice* package in R *mice* (Version 3.18.0; van Buuren and Groothuis-Oudshoorn, 2011). One response characterized by frequent use of “sometimes,” suggesting limited engagement, is excluded. The final sample comprises 76 pre-test responses and 41 post-test responses. Pre- and post-test data are merged using legal gender, mathematics class ID, problem-solving group ID, and prior mathematics grade, yielding 37 paired responses and 43 unpaired responses. Two responses, one reporting a failing grade F and one with missing prior grade information, are excluded from the regression analyses.

After imputing missing values, unidimensional IRT factor models are estimated for the latent variables of mathematics self-beliefs and student engagement, and corresponding factor scores are derived. Factor scores are derived from the posterior distributions using Maximum A Posteriori estimation and summarized using the median. In addition, sum scores are calculated for each latent variable to enable comparison with the IRT based scores. The correlations between the sum scores and the IRT based scores consistently exceed 0.90, indicating that the IRT factor scores perform at least as well as the corresponding sum scores.

### 6.1 Descriptive statistics and correlations

Differences between test scores (T1 and T2) are estimated using Bayesian analysis with participant values treated as random effects. These differences are generally small across variables, with somewhat larger but still uncertain changes observed for cognitive engagement (COE) and agentic engagement (AGE). The distributions of the factor scores (see Table 2) indicate variability across constructs, with evidence of outliers and skewness. To reduce the potential influence of outliers, robust estimation methods are applied in subsequent analyses.

**Table 2:** Summary of pre-test and post-test scores for all outcome measures.

Measure	Test diff	% ROPE	ICC	Factor score distribution	MSC	ASA	ICA	BEE	EME	COE	COD	AGE
GMS	0.01 [−0.17, 0.20]	63	0.76	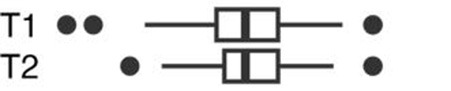	0.70^‡^(0)	−0.53^‡^ (0)	−0.21 (22.5)	0.39^‡^ (0.9)	0.46^‡^ (0.1)	0.48^‡^ (0.1)	−0.52^‡^ (0)	0.30^*^ (5.6)
MSC	0.08 [−0.05, 0.20]	54	0.90	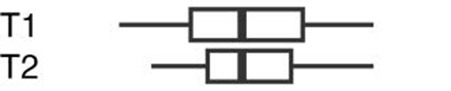		−0.35^*^ (3.8)	−0.20 (23.7)	0.21 (21.1)	0.41^†^ (1.3)	0.47^‡^ (0.4)	−0.32^*^ (5.5)	0.11 (38.8)
ASA	−0.10 [−0.28, 0.06]	38	0.72	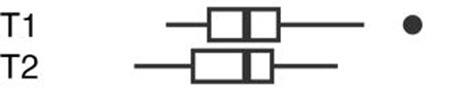			0.12 (35.3)	−0.19 (24.5)	−0.21 (18.5)	−0.16 (30.8)	0.66^‡^ (0)	−0.17 (28.2)
ICA	0.06 [−0.08, 0.19]	67	0.88	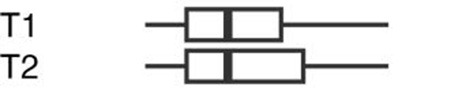				0.07 (40.3)	−0.01 (43.1)	−0.08 (42)	−0.02 (45)	−0.17 (28.9)
BEE	−0.13 [−0.27, 0.03]	32	0.87	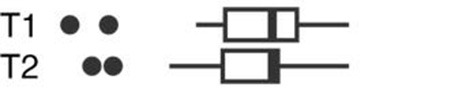					0.72^‡^ (0)	0.29^*^ (7.1)	−0.41^†^ (1.4)	0.51^‡^ (0.1)
EME	−0.04 [−0.21, 0.13]	68	0.86	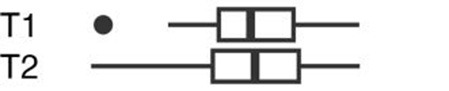						0.45^‡^ (0.4)	−0.37^†^ (2)	0.48^‡^ (0.2)
COE	0.22 [0.01, 0.43]	11	0.71	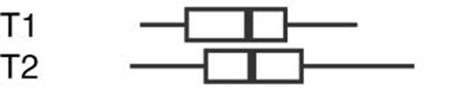							−0.21 (19.5)	0.26^*^ (11.2)
COD	−0.05 [−0.18, 0.07]	69	0.76	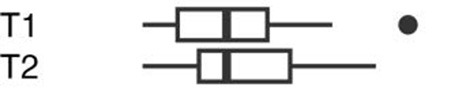								−0.30^*^ (7.5)
AGE	−0.22 [−0.39, −0.03]	8	0.76	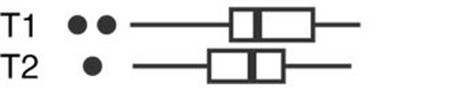								

*N* = 115. 95 % Credible Intervals (CI). Effects with a probability of direction (pd) levels: ^‡^ pd > 99.95 %,^†^ pd > 99.5 %, ^*^ pd > 97.5 %. Prior distribution generic weakly informative, Normal (0, 1). T1, pretest and T2, posttest.

Bayesian multilevel correlations are estimated to account for the dependency in the data. More specifically, these correlation coefficients are provided by the R package *brms* (Version 2.23.0; Bürkner, 2017, 2018, 2021), as Bayesian correlations are the residual correlations in an intercept-only multivariate model (participant value as random effect). To minimize the potential impact of outliers, a student model is employed to characterize the response distribution. In addition, the Intraclass Correlation Coefficients (ICC), which is estimated from the intercept-only model using the R package *performance* (Version 0.15.1; Lüdecke et al., 2021). The results indicate substantial variability among students, suggesting that the differences between them are pronounced. Overall, the ICC values indicated high internal consistency.

The results indicate that generalized mathematics self-efficacy (GMS) and mathematics self-concept (MSC) are strongly associated, while their associations with other variables differ. Generalized mathematics self-efficacy shows relatively consistent associations with most variables, whereas mathematics self-concept shows a more variable pattern. Specifically, when applying the threshold for significance within the ROPE of 2.5%, generalized mathematics self-efficacy shows statistically significant associations with all variables except in-class anxiety (ICA) and agentic engagement. Mathematics anxiety does not show statistically robust associations with all variables, and in-class anxiety is only weakly associated with other constructs. In addition, cognitive engagement and cognitive disengagement do not show a strong association, suggesting that these dimensions may capture distinct aspects of students' learning processes.

### 6.2 Associations with student engagement

To examine associations between mathematics self-beliefs, mathematics anxiety, and student engagement, a series of mixed-effects models were estimated using both frequentist and Bayesian approaches. To reduce the influence of outliers, parameter estimates for mixed models are generated using the R package *robustlmm* (Version 3.3.3; Koller, 2016). Because *p*-values are not directly available for this type of model (Koller, 2016), statistical significance is assessed using degrees of freedom estimated via the *lmer()* function in the R package *lmerTest* (Version 3.1.3; Kuznetsova et al., 2017). *p*-values are then calculated using the *pt()* distribution function in R (Version 4.5.1; R Core Team, 2023). To further reduce the influence of outliers, the models employ robust linear regression with a student's t-distribution for the response distribution.

The results indicate that prior mathematics achievement is associated with cognitive engagement primarily through its relation to generalized mathematics self-efficacy. For example, both lower and higher prior achievement levels are linked to differences in cognitive engagement that appear to be associated with corresponding differences in generalized mathematics self-efficacy.

**Figure 1:**
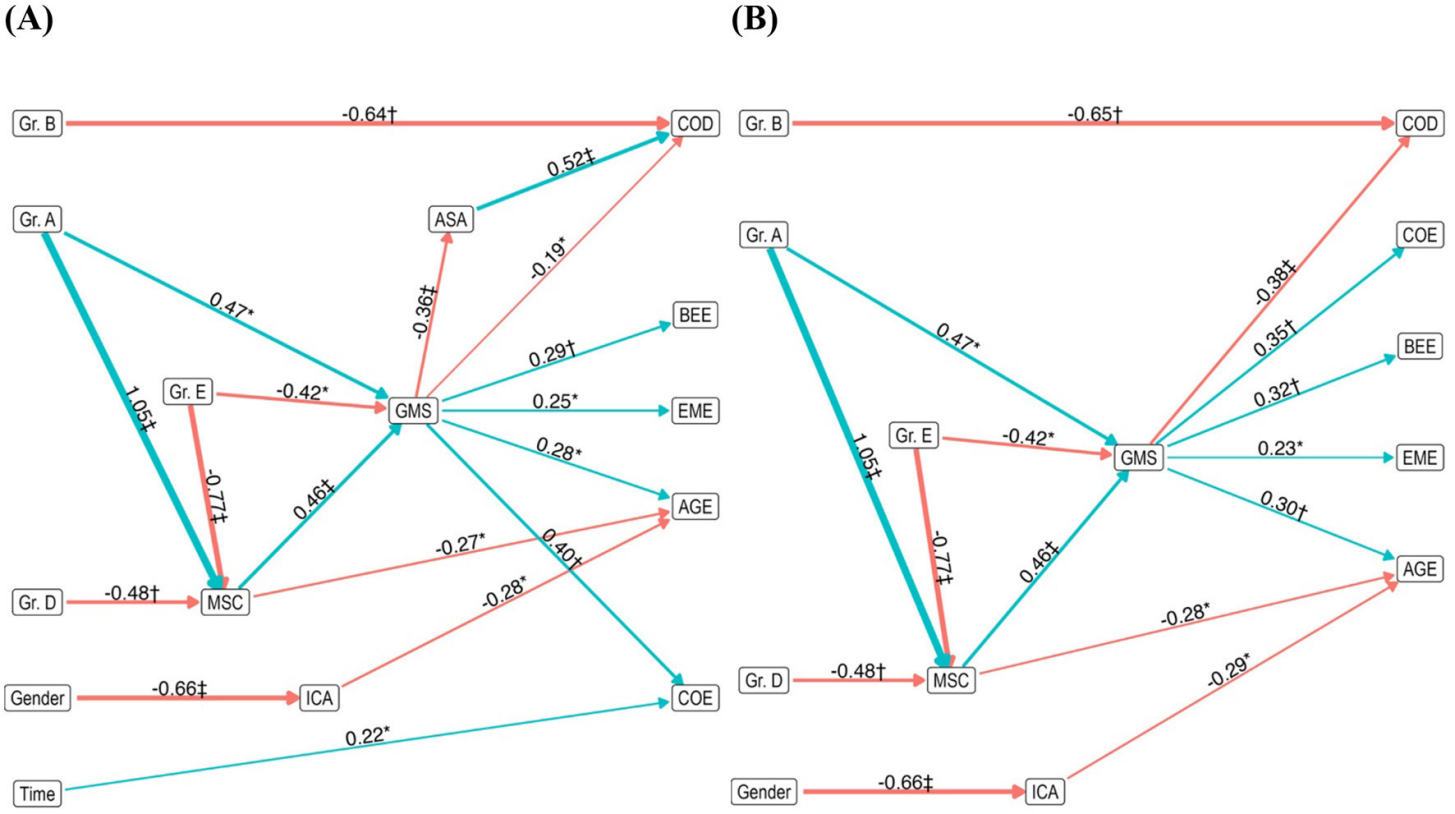
Path model. Only statistically supported paths are displayed for clarity. (**A**) With assignment anxiety (ASA); (**B**) Without assignment anxiety (ASA).

Table 3 shows the decomposition of effects from the path analysis after excluding non-significant relationships. The path models are presented in Figure 1. Complete regression results for all estimated models are provided in the Supplementary Material (Table 5). Figure 1A shows the model including assignment anxiety, whereas Figure 1B presents the corresponding model excluding assignment anxiety as a sensitivity analysis. Non-significant paths are retained in the analysis but omitted from the figure for clarity. Importantly, the figure depicts the full model rather than a recalculated reduced model excluding non-significant relationships. The directional paths reflect a theoretically informed ordering of variables rather than empirically established causal relationships.

**Table 3:** Engagement effects from path analysis excluding non-significant relationships.

Effect	(Intercept) β	*T*	Dir eff (pd)	%R	Ind eff (pd)	%R	Tot eff (pd)	%R	R^2^
On MSC	(0.03)								0.93 (0.48)
Of grade E	−0.77^‡^	−4.18	–**0.70 (100)**	**0.1**					
Of grade D	−0.48^†^	−2.97	–**0.38 (98)**	**6.5**					
Of grade A	1.05^‡^	4.72	**1.11 (100)**	**0.0**					
On ICA	(0.54^†^)								0.88 (0.24)
Of grade A	−0.44	−1.45	−0.50 (96)	**6.4**	−0.22 (97)	16.4	–**0.72 (100)**	**1.0**	
Of gender	−0.66^‡^	−3.51	–**0.63 (100)**	**0.2**	0.00 (58)	97.6	–**0.63 (100)**	**0.2**	
Of MSC	−0.20	−1.75	−0.20 (97)	16.0			−0.20 (97)	16.0	
On GMS	(−0.05)								0.78 (0.53)
Of grade E	−0.42^*^	−2.18	–**0.42 (98)**	**5.6**	–**0.33 (100)**	**1.4**	–**0.76 (100)**	**0.1**	
Of grade D	−0.15	−0.90	−0.14 (80)	32.4	–**0.18 (98)**	18.4	−0.33 (96)	10.1	
Of grade A	0.47^*^	2.05	0.48 (97)	**4.9**	**0.47 (100)**	**0.6**	**0.96 (100)**	**0.0**	
Of MSC	0.46^‡^	4.97	**0.47 (100)**	**0.0**	−0.01 (81)	99.6	**0.45 (100)**	**0.1**	
On ASA	(0.10)								0.83 (0.30)
Of MSC	0.10	0.98	0.11 (82)	43.1	–**0.20 (100)**	**5.7**	−0.09 (79)	47.2	
Of GMS	−0.36^‡^	−3.97	–**0.37 (100)**	**0.6**			–**0.37 (100)**	**0.6**	
On COD	(−0.01)								0.91 (0.53)
Of grade B	−0.64^†^	−3.38	–**0.65 (100)**	**0.9**	0.05 (64)	51.0	–**0.60 (99)**	**2.2**	
Of GMS	−0.19^*^	−2.45	–**0.22 (99)**	10.8	–**0.18 (100)**	**9.2**	–**0.40 (100)**	**0.3**	
Of ASA	0.52^‡^	7.40	**0.49 (100)**	**0.0**			**0.49 (100)**	**0.0**	
On COE	(−0.16)								0.79 (0.22)
Of grade E	0.43	1.45	0.37 (90)	12.9	–**0.33 (99)**	**5.2**	0.03 (54)	26.8	
Of grade A	−0.04	−0.11	−0.10 (61)	21.7	**0.47 (99)**	**2.9**	0.37 (87)	13.4	
Of time	0.22^*^	2.18	**0.22 (98)**	11.2	0.00 (53)	93.1	0.22 (97)	14.2	
Of MSC	0.26	1.78	0.25 (96)	13.0	**0.21 (100)**	**8.4**	**0.47 (100)**	**0.5**	
Of GMS	0.40^†^	2.99	**0.41 (100)**	**1.0**	−0.04 (83)	88.4	**0.37 (100)**	**1.7**	
On EME	(0.04)								0.89 (0.25)
Of GMS	0.25^*^	2.11	**0.27 (99)**	**8.4**	−0.01 (60)	97.0	**0.26 (99)**	**9.0**	
On BEE	(0.26)								0.91 (0.25)
Of grade B	0.46	1.74	0.46 (95)	**7.1**	−0.04 (67)	63.4	0.41 (92)	10.3	
Of gender	−0.40	−1.89	–**0.49 (99)**	**3.8**	−0.02 (59)	69.7	–**0.51 (99)**	**2.8**	
Of GMS	0.29^†^	2.80	**0.29 (100)**	**4.0**	0.03 (80)	94.5	**0.32 (100)**	**1.7**	
On AGE	(0.33)								0.79 (0.19)
Of gender	−0.37	−1.85	−0.39 (97)	**7.1**	0.20 (97)	16.9	−0.18 (81)	25.0	
Of MSC	−0.27^*^	−2.07	–**0.30 (98)**	**7.5**	**0.19 (99)**	13.1	−0.10 (78)	42.9	
Of ICA	−0.28^*^	−2.52	–**0.27 (99)**	**7.5**	0.02 (69)	96.9	–**0.25 (98)**	10.6	
Of GMS	0.28^*^	2.31	**0.32 (99)**	**4.7**	0.02 (71)	94.8	**0.34 (100)**	**2.7**	

*N* = 115. Significance levels: ^‡^ *p* < 0.001,^†^ *p* < 0.01, ^*^ *p* < 0.05 (two-tailed). Effects with a probability of direction (pd) above 97.5 % and a percentage in ROPE below 10% appear in bold. Dummy coding: gender (0, Female; 1, Male), and prior math grade (reference group C). Prior distribution weakly informative, Normal (0, 10).

To determine the extent to which the predictors account for variability in each model, Nakagawa's marginal and conditional *R*^2^ values are estimated for mixed models using the R package *performance* (Version 0.15.1; Lüdecke et al., 2021). The conditional *R*^2^ value incorporates both random and fixed effects, while the marginal *R*^2^ value (shown in parentheses) accounts only for the variance attributed to fixed effects. For instance, the independent variables account for 79% of the variability in the dimensions of cognitive engagement and agentic engagement, as indicated by the conditional *R*^2^ value.

The results presented in Table 3 indicate only minor differences in the parameter estimates obtained from the robust linear regression (see β) compared with those derived from the Bayesian approach, reported in the columns labeled *Dir eff (pd), Ind eff (pd)*, and *Tot eff (pd)*. The table also reports the probability of direction (pd) and percentage in ROPE (%R). Parameter estimates (β) from both the linear mixed model and the Bayesian model are similar in magnitude; however, the frequentist estimate is not statistically significant, whereas the Bayesian estimate has a probability of direction (pd) exceeding 97.5%. For several parameters, effects are considered practically meaningful when pd values are high (97–100%) and the proportion within the ROPE is low (below 10%). These effects, presumed to be of practical importance, appear in bold in Table 3.

The results show that prior mathematics achievement is associated with cognitive engagement primarily through its relation to generalized mathematics self-efficacy. For instance, for grade E, the effect on cognitive engagement is fully consistent with a mediating role of generalized mathematics self-efficacy [Median = −0.33, 95% CI (−0.66, −0.05)], with a 98.8% probability of being negative. Similarly, grade A influences cognitive engagement through generalized mathematics self-efficacy [Median = 0.47, 95% CI (0.08, 0.89)], with a 99.1% probability of being positive. Furthermore, assignment anxiety shows patterns consistent with an intermediary role in the association between generalized mathematics self-efficacy and cognitive disengagement [Median = −0.18, 95% CI (−0.31, −0.07)].

Generalized mathematics self-efficacy is negatively associated with cognitive disengagement [Median = −0.22, 95% CI (−0.40, −0.03)] and positively associated with several engagement dimensions. In addition, assignment anxiety is positively associated with cognitive disengagement and shows patterns consistent with an intermediary role in the association between generalized mathematics self-efficacy and disengagement.

Mathematics self-concept shows more limited associations with engagement. A notable association is observed with agentic engagement [Median = −0.30, 95% CI (−0.58, −0.03)], where mathematics self-concept is related both directly and indirectly through its associations with generalized mathematics self-efficacy and in-class anxiety [Median = 0.19, 95% CI (0.04, 0.39)]. These patterns suggest a more complex relationship between mathematics self-concept and engagement compared to generalized mathematics self-efficacy.

As shown in Figure 1A, generalized mathematics self-efficacy shows consistent associations with all dimensions of engagement and is also related to mathematics anxiety. Mathematics self-concept shows more limited associations, primarily with agentic engagement. The observed patterns suggest that associations between prior achievement and engagement may be linked to mathematics self-beliefs, particularly generalized mathematics self-efficacy. In addition, assignment anxiety is associated with cognitive disengagement. The relatively low reliability of the assignment anxiety scale, particularly at post-test, should be considered when interpreting this association. However, the corresponding model without assignment anxiety (Figure 1B) shows a similar overall pattern, suggesting that the observed associations are robust to the exclusion of this variable.

Comparisons between total effects and correlations indicate that the associations between generalized mathematics self-efficacy and emotional engagement, as well as between assignment anxiety and cognitive disengagement, are largely reflected in shared covariation. The results further show that indirect associations linked to prior achievement are more pronounced than the corresponding direct associations, whereas mathematics self-concept exhibits comparatively weaker associations. Taken together, these findings are broadly consistent with the proposed path model, in that mathematics self-beliefs are associated with emotional responses, which in turn are associated with different dimensions of engagement.

## 7 Discussion

The present study examined associations between mathematics self-beliefs, mathematics anxiety, and multiple dimensions of student engagement in the context of collaborative problem-solving. Overall, generalized mathematics self-efficacy showed the most consistent associations with engagement, whereas mathematics self-concept and mathematics anxiety displayed more variable and selective patterns. Although two measurement occasions were included, time was modeled as a predictor capturing differences between observations rather than as a basis for longitudinal inference. The findings should therefore be interpreted as associational rather than temporal relationships.

From a Control-Value Theory perspective (Pekrun, 2006), these findings are consistent with the assumption that competence-related self-beliefs reflect control-related appraisals that are associated with achievement emotions, which in turn are related to engagement. This interpretation is also in line with broader motivational frameworks linking self-beliefs to engagement in classroom contexts (Connell and Wellborn, 1991; Skinner and Belmont, 1993; Skinner et al., 2008, 2009). However, given the observational design and limited temporal separation between variables, these relations should be interpreted as associational rather than causal.

A central finding is that generalized mathematics self-efficacy shows strong and consistent associations with all dimensions of engagement. This aligns with previous research highlighting the role of self-efficacy beliefs in students' engagement and academic behavior (Zimmerman, 2000; Linnenbrink and Pintrich, 2003). One possible interpretation is that generalized mathematics self-efficacy reflects a prospective sense of competence that is closely aligned with students' active involvement in learning activities. In contrast, mathematics self-concept, as a more retrospective and evaluative construct (Marsh et al., 2019), shows weaker and more selective associations with engagement. This pattern suggests that future-oriented competence beliefs may be more directly related to ongoing engagement than broader self-evaluations of ability.

At the same time, the distinction between mathematics self-concept and generalized mathematics self-efficacy remains complex. Although the constructs are theoretically differentiated, their strong empirical association indicates partial overlap in measurement. This overlap may help explain why mathematics self-concept shows limited unique associations with engagement once generalized mathematics self-efficacy is included in the model. These findings highlight the importance of carefully distinguishing between different types of self-beliefs in future research, both conceptually and empirically.

The role of mathematics anxiety appears to be more nuanced. In the present study, mathematics anxiety is primarily associated with cognitive disengagement, consistent with previous research linking anxiety to reduced engagement and avoidance-related tendencies (Archambault et al., 2022). From a Control-Value Theory perspective, this pattern may reflect how negative achievement emotions are related to cognitive resources and motivational processes. For example, anxiety has been suggested to interfere with working memory and may be associated with a reduced tendency to engage in deeper learning strategies. At the same time, in-class anxiety shows relatively weak associations with other variables, suggesting that its role may be context-dependent. This interpretation is consistent with research indicating that achievement emotions vary across situations and task demands (Pekrun, 2006).

An additional finding concerns the distinction between cognitive engagement and cognitive disengagement. The lack of a strong association between these constructs suggests that disengagement may not simply represent the absence of engagement, but rather a qualitatively distinct process. This interpretation is consistent with multidimensional models of engagement that treat engagement and disaffection as partially independent dimensions (Skinner et al., 2008, 2009; Jang et al., 2016). In the present study, mathematics anxiety, particularly assignment anxiety, is more strongly associated with cognitive disengagement than with engagement measures, indicating that disengagement and engagement are not uniformly related across variables.

The results further suggest that associations between prior achievement and engagement may be linked to self-beliefs, particularly generalized mathematics self-efficacy. This is consistent with research showing that prior achievement is closely related to competence-related self-beliefs (Holenstein et al., 2022; Bergqvist, 2024) and with evidence of reciprocal relations between achievement and self-concept over time (Marsh, 1990; Marsh and Yeung, 1997). However, these interpretations remain tentative and should be examined in future research.

The negative association between mathematics self-concept and agentic engagement represents a theoretically interesting finding. One possible interpretation is that students with a stronger, more stable sense of competence may feel less need to actively influence instruction or seek support, whereas students with lower self-concept may engage more as a way of regulating their learning environment. At the same time, this association appears to be partially linked to generalized mathematics self-efficacy and in-class anxiety, suggesting that multiple processes may be involved. This finding highlights the importance of distinguishing between different forms of engagement and their underlying motivational dynamics.

The observed associations between prior achievement and engagement appear to be linked to mathematics self-beliefs, particularly generalized mathematics self-efficacy. This is consistent with research showing that prior achievement is closely related to competence-related beliefs (Holenstein et al., 2022) and may indirectly relate to engagement through these beliefs. However, prior achievement was measured using self-reported grades and should therefore be interpreted as an approximate indicator rather than an objective measure.

The collaborative problem-solving context may also have influenced the observed patterns. Collaborative settings involve peer interaction, shared regulation of learning, and opportunities for social comparison, which may shape students' perceptions of competence and emotional experiences. These features are theoretically relevant within both SSMMD and CVT, as they may influence control appraisals and achievement emotions. However, collaboration was not directly measured in the present study, and its specific role in shaping mathematics self-beliefs, emotions, and engagement therefore remains unclear. Accordingly, the findings should be interpreted as reflecting associations within this instructional context rather than effects of collaboration *per se*.

### 7.1 Future directions and limitations

Several limitations should be acknowledged. First, the relatively small sample size limits the generalizability of the findings and the stability of parameter estimates. Although Bayesian methods and robust estimation techniques were used to mitigate these issues, they do not fully compensate for limited statistical power or potential model instability. While the analytical approach may appear complex relative to the sample size, each method was used to examine different aspects of the data (measurement, association, robustness). Importantly, conclusions are based on converging patterns across methods rather than reliance on any single model. In particular, the path analysis represents an examination of patterns of associations rather than a test of a fully specified causal model.

Second, the use of repeated observations from pre- and post-tests does not constitute a fully longitudinal design. Although time was included as a predictor, the study design does not allow for disentangling within-person change from between-person differences. Future research using fully longitudinal designs is needed to examine temporal ordering and potential causal relationships.

Third, measurement limitations should be acknowledged. The relatively low reliability of the assignment anxiety scale, particularly at post-test, limits the strength of conclusions involving this variable. At the same time, analyses excluding this variable yielded similar overall pattern for the remaining predictors, suggesting that the main findings are not dependent on its inclusion.

Finally, the study relies on self-reported data, including prior achievement, which may introduce bias. Future research should incorporate objective measures of achievement and consider multimethod approaches to assessing engagement and emotional processes.

### 7.2 Conclusion

This study contributes to the literature by examining mathematics self-beliefs and mathematics anxiety jointly in relation to multiple dimensions of student engagement within a collaborative learning context. The results indicate that competence-related self-beliefs, particularly generalized mathematics self-efficacy, show consistent associations with student engagement, whereas mathematics anxiety is more closely related to disengagement processes. These patterns are broadly in line with theoretical perspectives emphasizing the interplay between self-beliefs, achievement emotions, and engagement (Pekrun, 2006; Skinner et al., 2008, 2009). Taken together, the findings highlight the importance of distinguishing between different types of mathematics self-beliefs and emotional processes when examining student engagement in mathematics.

An interesting aspect of the findings is the differential role of closely related constructs. While generalized mathematics self-efficacy shows consistent associations across engagement dimensions, mathematics self-concept displays more limited and selective patterns, despite their empirical overlap. In addition, mathematics anxiety appears to be more strongly linked to cognitive disengagement than to engagement itself, supporting the view that disengagement constitutes a distinct process rather than simply the absence of engagement. These results point to the value of examining both engagement and disengagement, as well as distinguishing between prospective competence beliefs and retrospective self-evaluations.

Future research should build on these findings by employing larger samples and longitudinal designs that allow for a more precise examination of temporal ordering and potential causal dynamics among mathematics self-beliefs, mathematics anxiety, and student engagement.

## Data Availability

The datasets presented in this study can be found in online repositories. The names of the repository/repositories and accession number(s) can be found below: https://doi.org/10.5878/y87v-t432.
