# Supplementary material for: Generalized mathematics self-efficacy and student engagement: the role of self-concept and mathematics anxiety
Source: Front Psychol. 2026 Jun 15;17:1852588. doi: 10.3389/fpsyg.2026.1852588 (PMC13311082; doi:10.3389/fpsyg.2026.1852588)
Supplement: Supplementary file 1 [file Supplementary_file_1.docx]

**Table 4** Engagement items with their original wording presented in italics

|  |
| --- |
| **Behavioral Engagement (BEE)** |
| Jag lyssnar ordentligt. *When I'm in this class, I listen very carefully.* |
| Jag är uppmärksam. *I pay attention in this class.* |
| Jag försöker göra mitt bästa för att det ska gå bra för mig. *I try hard to do well in this class.* |
| Jag jobbar så hårt jag bara kan. *In this class, I work as hard as I can.* |
| **Emotional Engagement (EME)** |
| Jag tycker det är roligt på lektionen. *This class is fun.* |
| Jag gillar att lära mig nya saker under lektionen. *I enjoy learning new things in this class.* |
| Jag mår bra på lektionen. *When I'm in this class, I feel good.* |
| Jag är intresserad av det vi jobbar med på lektionen. *When we work on something in this class, I feel interested.* |
| Jag är engagerad i det vi jobbar med på lektionen. *When we work on something in this class, I get involved.* |
| **Cognitive Engagement (COE)** |
| Jag försöker beskriva matematiska begrepp i kursen med egna ord. *When reading for this class, I try to explain the key concepts in my own words.* |
| Jag försöker sammanfatta nya moment i kursen med egna ord. *When learning about a new topic in this course, I usually try to summarize it in my own words.* |
| Jag försöker koppla ihop det jag lär mig med det jag redan kan. *When reading for this class, I try to connect the ideas I am reading about with what I already know.* |
| Jag försöker skapa egna exempel som ska hjälpa mig att förstå matematiska begrepp. *When thinking about the concepts in this class, I try to generate examples to help me understand them better.* |
| **Cognitive Disengagement (COD)** |
| Jag vet inte vad jag ska plugga eller var jag ska börja. *In this course, I often find that I don't know what to study or where to start.* |
| Jag är osäker på hur jag ska plugga. *I'm not sure how to study for this course.* |
| Jag tycker det är svårt att plugga på ett tidseffektivt sätt. *In this course, I find it difficult to organize my study time effectively.* |
| Jag har svårt att komma på hur jag ska göra för att lära mig matematik *When I study for this course, I have trouble figuring out what to do to learn the material.* |
| **Agentic Engagement (AGE)** |
| Jag ser till att min lärare vet vad jag vill och behöver. *I let my teacher know what I need and want.* |
| Jag uttrycker mina åsikter och vad jag helst vill göra. *During this class, I express my preferences and opinions.* |
| Jag frågar läraren när jag behöver något.  *When I need something in this class, I'll ask the teacher for it.* |
| Jag ställer frågor för att lära mig bättre. *During class, I ask questions to help me learn.* |
| Jag ser till att min lärare vet vad jag tycker är intressant. *I let my teacher know what I am interested in.* |

**Table** **5** Complete regression results

| Effect | (Intercept)  β | t | Dir eff (pd) | %R | Ind eff (pd) | %R | Tot eff (pd) | %R | $R^{2}$ |
| --- | --- | --- | --- | --- | --- | --- | --- | --- | --- |
| On MSC | (0.03) |  |  |  |  |  |  |  | 0.93(0.48) |
| of grade E | -0.77‡ | -4.18 | **-0.70(100)** | **0.1** |  |  |  |  |  |
| of grade D | -0.48† | -2.97 | **-0.38(98)** | **6.5** |  |  |  |  |  |
| of grade B | 0.14 | 0.70 | 0.19(78) | 23.5 |  |  |  |  |  |
| of grade A | 1.05‡ | 4.72 | **1.11(100)** | **0.0** |  |  |  |  |  |
| of gender | 0.05 | 0.33 | 0.04(60) | 46.1 |  |  |  |  |  |
| of time | 0.09 | 1.67 | 0.07(84) | 68.5 |  |  |  |  |  |
| On ICA | (0.54†) |  |  |  |  |  |  |  | 0.88(0.24) |
| of grade E | -0.12 | -0.50 | -0.12(70) | 29.6 | 0.13(97) | 34.7 | 0.02(53) | 34.2 |  |
| of grade D | -0.13 | -0.61 | -0.16(79) | 28.8 | 0.07(95) | 70.4 | -0.08(67) | 35.7 |  |
| of grade B | 0.15 | 0.55 | 0.11(68) | 28.1 | -0.03(76) | 85.1 | 0.08(61) | 29.3 |  |
| of grade A | -0.44 | -1.45 | -0.50(96) | **6.4** | -0.22(97) | 16.4 | **-0.72(100)** | **1.0** |  |
| of gender | -0.66‡ | -3.51 | **-0.63(100)** | **0.2** | 0.00(58) | 97.6 | **-0.63(100)** | **0.2** |  |
| of time | 0.07 | 1.00 | 0.07(84) | 65.3 | -0.01(82) | 100.0 | 0.06(79) | 70.6 |  |
| of MSC | -0.20 | -1.75 | -0.20(97) | 16.0 |  |  | -0.20(97) | 16.0 |  |
| On GMS | (-0.05) |  |  |  |  |  |  |  | 0.78(0.53) |
| of grade E | -0.42* | -2.18 | **-0.42(98)** | **5.6** | **-0.33(100)** | **1.4** | **-0.76(100)** | **0.1** |  |
| of grade D | -0.15 | -0.90 | -0.14(80) | 32.4 | **-0.18(98)** | 18.4 | -0.33(96) | 10.1 |  |
| of grade B | -0.09 | -0.41 | -0.12(69) | 28.6 | 0.09(78) | 48.2 | -0.02(53) | 29.2 |  |
| of grade A | 0.47* | 2.05 | 0.48(97) | **4.9** | **0.47(100)** | **0.6** | **0.96(100)** | **0.0** |  |
| of gender | 0.13 | 0.93 | 0.12(78) | 38.2 | -0.04(65) | 68.0 | 0.08(69) | 42.0 |  |
| of time | -0.01 | -0.09 | -0.02(58) | 70.2 | 0.04(88) | 95.9 | 0.02(58) | 67.5 |  |
| of MSC | 0.46‡ | 4.97 | **0.47(100)** | **0.0** | -0.01(81) | 99.6 | **0.45(100)** | **0.1** |  |
| of ICA | 0.06 | 0.69 | 0.09(83) | 53.9 |  |  | 0.09(83) | 53.9 |  |
| On ASA | (0.10) |  |  |  |  |  |  |  | 0.83(0.30) |
| of grade E | 0.06 | 0.26 | 0.08(63) | 32.4 | 0.07(71) | 52.5 | 0.14(73) | 27.5 |  |
| of grade D | 0.17 | 0.92 | 0.18(82) | 27.1 | 0.00(52) | 73.1 | 0.17(79) | 27.4 |  |
| of grade B | 0.04 | 0.18 | 0.03(56) | 31.8 | 0.07(77) | 55.2 | 0.11(66) | 27.4 |  |
| of grade A | -0.34 | -1.30 | -0.31(87) | 15.3 | -0.12(78) | 34.9 | -0.44(95) | **8.3** |  |
| of gender | -0.20 | -1.23 | -0.26(93) | 16.0 | -0.12(92) | 41.5 | **-0.38(98)** | **5.0** |  |
| of time | -0.11 | -1.48 | -0.10(88) | 47.3 | 0.02(71) | 95.9 | -0.08(80) | 54.9 |  |
| of MSC | 0.10 | 0.98 | 0.11(82) | 43.1 | **-0.20(100)** | **5.7** | -0.09(79) | 47.2 |  |
| of ICA | 0.10 | 1.08 | 0.13(91) | 36.8 | -0.03(83) | 95.8 | 0.10(82) | 46.5 |  |
| of GMS | -0.36‡ | -3.97 | **-0.37(100)** | **0.6** |  |  | **-0.37(100)** | **0.6** |  |
| On COD | (-0.01) |  |  |  |  |  |  |  | 0.91(0.53) |
| of grade E | 0.02 | 0.13 | -0.02(53) | 37.0 | 0.09(75) | 42.2 | 0.08(63) | 31.6 |  |
| of grade D | 0.04 | 0.27 | -0.02(55) | 45.5 | 0.11(82) | 44.1 | 0.08(67) | 36.8 |  |
| of grade B | -0.64† | -3.38 | **-0.65(100)** | **0.9** | 0.05(64) | 51.0 | **-0.60(99)** | **2.2** |  |
| of grade A | -0.35 | -1.54 | -0.30(86) | 16.1 | -0.16(80) | 28.6 | -0.46(94) | **8.2** |  |
| of gender | 0.13 | 0.90 | 0.21(90) | 22.0 | -0.12(86) | 41.4 | 0.09(69) | 38.0 |  |
| of time | 0.03 | 0.52 | 0.01(58) | 84.4 | -0.05(81) | 84.2 | -0.03(66) | 73.1 |  |
| of MSC | 0.07 | 0.80 | 0.06(72) | 59.1 | -0.04(69) | 72.2 | 0.02(57) | 59.1 |  |
| of ICA | -0.09 | -1.12 | -0.05(74) | 65.9 | 0.04(78) | 83.7 | -0.01(54) | 66.4 |  |
| of GMS | -0.19* | -2.45 | **-0.22(99)** | 10.8 | **-0.18(100)** | **9.2** | **-0.40(100)** | **0.3** |  |
| of ASA | 0.52‡ | 7.40 | **0.49(100)** | **0.0** |  |  | **0.49(100)** | **0.0** |  |
| On COE | (-0.16) |  |  |  |  |  |  |  | 0.79(0.22) |
| of grade E | 0.43 | 1.45 | 0.37(90) | 12.9 | **-0.33(99)** | **5.2** | 0.03(54) | 26.8 |  |
| of grade D | 0.22 | 0.88 | 0.18(76) | 24.2 | -0.13(87) | 37.9 | 0.05(58) | 29.1 |  |
| of grade B | -0.05 | -0.15 | -0.08(60) | 25.6 | 0.00(50) | 54.7 | -0.08(59) | 23.8 |  |
| of grade A | -0.04 | -0.11 | -0.10(61) | 21.7 | **0.47(99)** | **2.9** | 0.37(87) | 13.4 |  |
| of gender | -0.04 | -0.18 | -0.02(54) | 35.7 | 0.06(72) | 54.5 | 0.05(59) | 34.9 |  |
| of time | 0.22* | 2.18 | **0.22(98)** | 11.2 | 0.00(53) | 93.1 | 0.22(97) | 14.2 |  |
| of MSC | 0.26 | 1.78 | 0.25(96) | 13.0 | **0.21(100)** | **8.4** | **0.47(100)** | **0.5** |  |
| of ICA | -0.08 | -0.63 | -0.05(68) | 54.8 | 0.05(88) | 85.1 | -0.01(52) | 57.2 |  |
| of GMS | 0.40† | 2.99 | **0.41(100)** | **1.0** | -0.04(83) | 88.4 | **0.37(100)** | **1.7** |  |
| of ASA | 0.14 | 1.14 | 0.11(83) | 41.5 |  |  | 0.11(83) | 41.5 |  |
| On EME | (0.04) |  |  |  |  |  |  |  | 0.89(0.25) |
| of grade E | -0.31 | -1.11 | -0.32(87) | 14.9 | -0.12(86) | 38.9 | -0.44(95) | **8.3** |  |
| of grade D | -0.42 | -1.75 | -0.40(95) | **9.0** | -0.04(71) | 70.0 | -0.45(96) | **6.8** |  |
| of grade B | 0.35 | 1.20 | 0.33(86) | 14.9 | -0.02(59) | 71.4 | 0.31(84) | 16.1 |  |
| of grade A | 0.58 | 1.63 | 0.33(82) | 14.7 | 0.11(74) | 37.0 | 0.44(90) | 10.2 |  |
| of gender | -0.01 | -0.06 | 0.00(50) | 35.0 | 0.00(52) | 71.2 | 0.00(50) | 35.0 |  |
| of time | -0.04 | -0.51 | -0.04(69) | 69.1 | 0.00(51) | 98.3 | -0.05(69) | 67.0 |  |
| of MSC | 0.02 | 0.12 | 0.01(52) | 53.0 | 0.12(95) | 41.8 | 0.13(84) | 38.0 |  |
| of ICA | 0.07 | 0.56 | 0.05(67) | 56.6 | 0.02(79) | 96.6 | 0.08(74) | 50.8 |  |
| of GMS | 0.25* | 2.11 | **0.27(99)** | **8.4** | -0.01(60) | 97.0 | **0.26(99)** | **9.0** |  |
| of ASA | 0.05 | 0.44 | 0.03(60) | 63.0 |  |  | 0.03(60) | 63.0 |  |
| On BEE | (0.26) |  |  |  |  |  |  |  | 0.91(0.25) |
| of grade E | -0.32 | -1.23 | -0.23(80) | 19.8 | -0.06(68) | 53.5 | -0.29(85) | 16.9 |  |
| of grade D | -0.21 | -0.98 | -0.16(77) | 26.9 | -0.03(63) | 68.8 | -0.20(81) | 24.4 |  |
| of grade B | 0.46 | 1.74 | 0.46(95) | **7.1** | -0.04(67) | 63.4 | 0.41(92) | 10.3 |  |
| of grade A | 0.33 | 1.02 | 0.14(66) | 21.3 | -0.03(57) | 42.6 | 0.11(62) | 22.4 |  |
| of gender | -0.40 | -1.89 | **-0.49(99)** | **3.8** | -0.02(59) | 69.7 | **-0.51(99)** | **2.8** |  |
| of time | -0.13 | -1.82 | -0.12(94) | 39.3 | 0.00(55) | 98.2 | -0.12(92) | 41.5 |  |
| of MSC | -0.13 | -1.08 | -0.13(83) | 37.3 | 0.10(92) | 50.2 | -0.03(58) | 55.7 |  |
| of ICA | 0.13 | 1.23 | 0.12(87) | 40.0 | 0.01(64) | 98.4 | 0.14(88) | 36.1 |  |
| of GMS | 0.29† | 2.80 | **0.29(100)** | **4.0** | 0.03(80) | 94.5 | **0.32(100)** | **1.7** |  |
| of ASA | -0.07 | -0.76 | -0.09(80) | 51.7 |  |  | -0.09(80) | 51.7 |  |
| On AGE | (0.33) |  |  |  |  |  |  |  | 0.79(0.19) |
| of grade E | -0.22 | -0.83 | -0.24(79) | 19.1 | 0.10(75) | 42.8 | -0.14(68) | 23.0 |  |
| of grade D | -0.18 | -0.80 | -0.19(80) | 24.1 | 0.09(79) | 50.4 | -0.10(64) | 28.4 |  |
| of grade B | 0.35 | 1.27 | 0.35(88) | 14.1 | -0.12(82) | 39.7 | 0.22(75) | 19.0 |  |
| of grade A | 0.17 | 0.51 | 0.05(56) | 23.1 | -0.02(55) | 38.4 | 0.02(52) | 24.0 |  |
| of gender | -0.37 | -1.85 | -0.39(97) | **7.1** | 0.20(97) | 16.9 | -0.18(81) | 25.0 |  |
| of time | -0.16 | -1.75 | -0.18(95) | 21.4 | -0.03(78) | 88.9 | -0.21(97) | 14.3 |  |
| of MSC | -0.27* | -2.07 | **-0.30(98)** | **7.5** | **0.19(99)** | 13.1 | -0.10(78) | 42.9 |  |
| of ICA | -0.28* | -2.52 | **-0.27(99)** | **7.5** | 0.02(69) | 96.9 | **-0.25(98)** | 10.6 |  |
| of GMS | 0.28* | 2.31 | **0.32(99)** | **4.7** | 0.02(71) | 94.8 | **0.34(100)** | **2.7** |  |
| of ASA | -0.06 | -0.52 | -0.06(71) | 56.3 |  |  | -0.06(71) | 56.3 |  |
| Note: N = 115. Significance levels: ‡ p < 0.001, † p < 0.01, * p < 0.05 (two-tailed). Effects with a probability of direction (pd) above 97.5 % and a percentage in ROPE below 10 % appear in bold. Dummy coding: Gender (0 = Female, 1 = Male), and prior math grade (reference group C). Prior distribution weakly informative = Normal(0,10). | | | | | | | | | |
